# A Novel Needle for Sutures

**Published:** 1860-10

**Authors:** 


*A Novel Needle for Sutures*, has been invented by Prof. P. F. Eve, to carry wire or silk, principally the former. It is a hollow needle, with an aperture just before its point, upon the inner curved surface, for the egress of the wire after the edges of the wound have been punctured by the needle. It is mounted upon a handle, the lower end, or eye, has the opening for the wire upon its under surface, so that the needle is thrust through the parts. The wire being straight, with its point within the needle, is now pushed a short distance through, and the needle withdrawn, leaving the wire in its place. It is now cut off, so as to leave an end protruding from either edge, and twisted or bent, as the case may require, care being had to prevent the sharp ends from wounding the surrounding parts. Platina wire would prove better than the silver, which is commonly used, from its being softer and less resisting.

It is a simple and ingenious improvement in the ordinary needle—very useful in staphyloraphy and other operations of the dentist. B.

